# SIRT3 deacetylase activity confers chemoresistance in AML via regulation of mitochondrial oxidative phosphorylation

**DOI:** 10.1111/bjh.16044

**Published:** 2019-06-24

**Authors:** Jiao Ma, Bin Liu, Dan Yu, Yong Zuo, Rong Cai, Jianmin Yang, Jinke Cheng

**Affiliations:** ^1^ Department of Biochemistry and Molecular Cell Biology Shanghai Jiaotong University School of Medicine Shanghai China; ^2^ Department of Haematology Changhai Hospital of the Second Military Medical University Shanghai China; ^3^ Department of Respiratory Medicine The People's Liberation Army General Hospital Shanghai China

**Keywords:** SIRT3, ROS, SOD2 acetylation, chemoresistance, AML

## Abstract

Acute myeloid leukaemia (AML) cells possess metabolism profiles, such as higher rates of oxidative phosphorylation and dependence on fatty acid oxidation for survival, and are dependent on the sophisticated regulation of reactive oxygen species (ROS) generation for survival, drug resistance and stemness maintenance. We found that sensitivity of primary AML cells to cytarabine correlated with SOD2 acetylation and the ability of the drug to induce mitochondrial ROS. The SOD2 deacetylase, SIRT3, protected AML cells from chemotherapy as shown by inhibited apoptosis via inhibited drug‐induced production of mitochondrial ROS. SIRT3 significantly decreased nicotinamide adenine dinucleotide phosphate (NADP)/reduced NADP ratio and increased reduced glutathione/oxidized glutathione ratio. Furthermore, SIRT3 enhanced oxidative phosphorylation (OxPhos) in AML cells under both basic and cytarabine‐treated conditions. A xenograft mouse model showed that SIRT3 overexpressing AML cells and patient‐derived xenograft mice bearing high SIRT3 deacetylase activity were more resistant to chemotherapy *in vivo*. SIRT3 inhibitor displayed synergy with cytarabine to ablate AML cells *in vitro* and in mouse models. Taken together, our study showed that SIRT3 is capable of reprograming mitochondrial metabolism towards OxPhos and downregulating ROS generation, which contribute to the chemoresistance of AML cells. SIRT3 can be utilized as a potential therapeutic target to improve the anti‐leukaemic efficacy of standard chemotherapeutic agents for AML.

The standard induction regimen for acute myeloid leukaemia (AML) has barely changed in over three decades: it is known as “7 + 3” regimen, with cytarabine (Ara‐C) 100 ~ 200 mg/m^2^/day for 7 consecutive days, and daunorubicin 60 mg/m^2^/day on the first 3 days (Dombret & Gardin, 2016). Although over 80% of younger patients receiving such treatment may achieve complete remission (CR), almost half of them will relapse, with the median 5‐year overall survival (OS) as low as 25% (Dohner *et al*, 2017). And the situation is even worse in young adults (Nasir *et al*, 2017). The high relapse rate and poor clinical outcome in AML make chemoresistance the most important and primary goal to improve the therapeutic efficacy and survival for these patients.

A variety of potential mechanisms involved in the development of chemoresistance in AML have been identified in the past few decades. These include drug efflux by transporters like MRD1, detoxification enzymes that defend cell against genotoxic attack by drugs, and the bone marrow niche that controls the living status of leukaemia cells under its hypoxic environment and limits the access of chemotherapeutic agents to leukaemia cells, allowing maintenance of minimal residual disease after treatment, and eventually leading to relapse of the disease (Zhou *et al*, 2016; Dohner *et al*, 2017). Furthermore, both Ara‐C and anthracyclines in the front line of standard induction therapy induce AML cell death by intercalating in the DNA strands and interfering with DNA synthesis. However, leukaemia‐initiating cells, or leukaemic stem cells (LSCs), the major cause of AML, constitute a rare population in the haematopoietic system capable of proliferating and differentiating into leukaemic blasts during leukemogenesis, and giving rise to relapse in patients after chemotherapy. They remain mostly quiescent in the cell cycle, allowing them to escape from the attack of such chemotherapeutic agents that are usually effective in rapidly proliferating cells (Bruserud *et al*, 2017).

It is widely accepted that cancer cells with mitochondrial deficiency are thought to rely more on glycolysis for ATP supply and different ROS homeostasis for survival against drugs (Liberti & Locasale, 2016). Increasing data have shown that metabolic reprogramming, which is thought to be driven by abnormalities in nutrient‐sensing pathways, including AMPK, mTOR, PI3K/AKT and their crosstalk to regulate ROS, is a key feature of transformed cells, and is essential for tumour cell proliferation (Caino & Altieri, 2016; Zhao *et al*, 2017). In addition to being the major sites for biosynthesis and energy supply, mitochondria initiate apoptotic signalling in AML cells triggered by chemotherapeutic agents, which includes anti‐apoptotic BCL2, BCL‐xL (also termed BCL2L1) and MCL1, and pro‐apoptotic BAX, BAK (BAK1) and cytochrome c, as well as ROS (Indran *et al*, 2011). Induction of ROS is a critical event regulating AML apoptosis induced by chemotherapeutic agents, such as arsenic trioxide, parthenolide (PTL) and Ara‐C (Guzman *et al*, 2005; Schimmer, 2017; Mesbahi *et al*, 2018). Sophisticated regulation of ROS level is important for both cell apoptosis and cellular homeostasis, especially for LSCs. However, the reports regarding ROS level in cancer cells, including AML blasts and stem cells, are controversial. Low levels of ROS are essential for the maintenance of stemness in LSCs, partly due to the hypoxic bone marrow niche, alongside the overexpression of BCL2 (Lagadinou *et al*, 2013). BCL2 inhibition downregulated oxidative phosphorylation (OxPhos) and displayed selective toxicity to quiescent LSCs *in vitro* and in patient‐derived xenograft mice (Lagadinou *et al*, 2013). Furthermore, a clinical study (NCT02203773) demonstrated that combination of BCL2 inhibitor venetoclax, an oral BCL2 inhibitor, with azacytidine or decitabine resulted in higher CR rate and OS (Pollyea *et al*, 2018; DiNardo *et al*, 2019). Howerver, Farge *et al *(2017) reported that Ara‐C‐resistant pre‐existing and persisting cells displayed high OxPhos status and elevated ROS levels. Therefore, targeting mitochondrial metabolism induced an energetic shift to low OxPhos, resulting in enhanced anti‐leukaemic efficacy of Ara‐C (Farge *et al*, 2017).

In AML cells, ROS is predominantly generated by mitochondria, with the involvement of various metabolic enzymatic systems, such as the mitochondrial electron transport chain, cytochrome P450 enzymes, lipoxygenases, cyclooxygenases, the reduced nicotinamide adenine dinucleotide phosphate (NADPH) oxidase complex, xanthine oxidase, peroxisomal enzymes, thymidine phosphorylase etc (Fatehi‐Hassanabad *et al*, 2010). It has been documented that post‐translational modifications, including phosphorylation, acetylation, succinylation, ubiquitination and SUMOylation among others, for a variety of mitochondrial proteins are capable of regulating the enzymatic activity of key metabolic enzymes (Figueroa‐Romero *et al*, 2009; Stram & Payne, 2016). One protein that mediates such post‐translational modifications of mitochondrial proteins is SIRT3, a NAD+‐dependent protein deacetylase that is reported to influence cellular metabolism and downregulate ROS generation by deacetylating mitochondrial anti‐oxidant enzymes (Chen *et al*, 2011). On the other hand, its de‐acetylase activity can also be regulated by post‐translational modifications (Liu *et al*, 2015). The mitochondrial targets of SIRT3 include superoxide dismutase 2 (SOD2) and isocitrate dehydrogenase 2 (IDH2), both of which are closely related to ROS generation and leukaemogenesis (Ward *et al*, 2010; Yu *et al*, 2012; Girerd *et al*, 2013; Liu *et al*, 2017). Given that sophisticated regulation of ROS production is required for the maintenance of leukaemic blasts and stem cells, we investigated the regulatory mechanism of mitochondrial ROS in chemoresistance of AML, which may not only contribute to a better understanding of SIRT3 in haematopoietic malignancies, but also provide the rationale of targeting SIRT3 to improve chemotherapy outcome.

## Materials and methods

See the Data S1 for additional details.

### Drug compounds and antibodies

Cytarabine (Ara‐C) and danorubicin were purchased from Sigma‐Aldrich (St. Louis, MI, USA). PTL and 3‐(1H‐1,2,3‐triazol‐4‐yl) pyridine (3‐TYP) were from Selleckchem (Houston, TX, USA). All compounds, except for *in vivo* studies, were reconstituted in dimethlysulfoxide, stored at 100‐mmol/l stock concentrations at −80°C, and used at the indicated doses suggested by the vendor. Flow cytometry antibodies, Alexa Fluor 647 Rabbit Anti‐Active caspase 3, PE‐Cy7 Mouse Anti‐Human CD38, APC‐H7 Mouse Anti‐Human CD45, and APC Mouse Anti‐Human CD34 were purchased from BD Pharmingen (San Jose, CA, USA). Immunoblotting antibodies, cleaved caspase 9, MCL1, BCL2, BAD, BAX, acetylated lysine and SIRT3 were purchased from Cell Signalling Technology (Danvers, MA, USA). Acetylated SOD2 and SOD2 antibodies were purchased from Abcam. ATPA (51) antibody was purchased from Santa Cruz Biotechnology (Dallas, TX, USA).

### Cell lines, primary cells and culture conditions

The AML cell lines Kasumi‐1, MV4‐11 MOLM‐13, U937, KG‐1 and THP‐1 were cultured in Iscove’s Modified Dulbecco’s Medium (IMDM) supplemented with 10–20% fetal bovine serum (FBS) and 100 µg/ml penicillin/streptomycin. All cells were maintained in a humidified 37°C/5% CO_2 _incubator. Primary AML cells were obtained from the Department of Haematology at Changhai Hospital after Institutional Review Board review and approval (#CHEC‐2018‐115). All primary cells were thawed and sub‐cultured as previously described.

### Mitochondria isolation

Aliquots (50 × 10^9^) of Vector control, SIRT3 or shSIRT3#3 lentiviral transduced AML cell pellets were harvested and washed with ice‐cold TD buffer (135 mmol/l NaCl, 5 mmol/l KCl, 25 mmol/l Tri‐HCl, PH 7·5) twice by centrifugation at 500 ***g*** for 10 min. Cells were then re‐suspended in 1 ml ice‐cold MS buffer [210 mmol/l mannitol, 70 mmol/l sucrose, 5 mmol/l Tris‐HCl, PH 7. 5, 1 mmol/l egtazic acid, 1 mmol/l phenylmethylsulfonyl fluoride (PMSF), 1 ug/ml Leupeptin, 10 ug/ml aprotinin, 1 mmol/l N‐Ethylmaleimide (NEM)], and homogenized approximate 45 times until more than 50% of the cells had died. Mitochondria were isolated by centrifugation at 10 000 ***g*** for 1 min and washed three times with ice‐cold MS buffer. Mitochondria fractions were then lysed with 1× radioimmunoprecipitation assy buffer (50 mmol/l Tris‐HCl, PH 7·5, 50 mmol/l NaCl, 0·3% Nonidet P‐40, 1 mmol/l PMSF, 1 µg/ml Leupeptin, 10 µg/ml aprotinin, 1 mmol/l NEM) for 30 min on ice, and harvested at 14 000 g for 10 min at 4°C.

### Total and mitochondrial ROS staining

CellROX and MitoSOX staining probes were purchased from Thermofisher Technology Inc. (Waltham, MA, USA), and AML cell lines or primary AML cells were stained according to instruction manual. Briefly, cells were treated with either Ara‐C or vehicle control for 48 h. Cells were probed with 25 µmol/l CellROX for 30 min at 37°C or 5 µmol/l MitoSOX for 10 min at 37°C. AML cells were then washed twice with fluorescence‐activated cell sorting (FACS) buffer, prior to subject to flow cytometry assay.

### Apoptosis assay

Vector control, wild type SIRT3 or shSIRT3 transduced AML cells were treated with either 1 µmol/l Ara‐C or vehicle control. Cells were then lysed and probed with a panel of apoptotic markers (antibodies section) for immunoblotting assay, or fixed/ permeabilised and stained with caspase 3 antibody (antibodies section) for flow cytometric analysis.

### Metabolism assays

#### Nicotinamide adenine dinucleotide phosphate (NADP)/reduced NADP (NADPH), reduced glutathione/oxidized glutathione (GSH/GSSG), Glucose uptake assays

NADP/NADPH (Abcam, Cambridge, UK), GSH/GSSG (Abcam), Glucose uptake (Promega, Madison, WI, USA) assays were carried according to manufacturers’ instructions.

#### Extracellular acidification rate and basal oxygen consumption rate

Oxygen consumption rate (OCR) and extracellular acidification rate (ECAR) assays were performed as per instruction manual (Seahorse Bioscience, Santa Clara, CA, USA). Briefly, AML cells were treated with either 1 µmol/l Ara‐C or vehicle control for 48 h. Cells were then seeded in duplicates at a density of 5 × 10^5^ in a XF96 cell culture microplate, which was pre‐coated with Corning® Cell‐Tak™ Cell and Tissue Adhesive (Corning Incorporated, New York, NY, USA) to allow adhesion of suspension cells. To test mitochondria respiration, sequential compound injections, including oligomycin A, carbonyl‐cyanide p‐trifluoromethoxyphenylhydrazone (FCCP), antimycin A and rotenone, were applied to the microplate after analyser calibration. To test glycolytic activity, glucose, oligomycin A and 2‐DG, were sequentially injected on the microplate followed by calibration step. Data was analysed by Wave 2.2.0 software (Agilent Technologies, Santa Clara, CA, USA).

### Drug synergy

AML cells were seeded into 96‐well plates at approximately 10 000 cells/well and allowed to grow for 24 h. Ara‐C (1 µmol), SIRT3 selective inhibitor 3‐TYP (10 µmol), or a combination of these two drugs were added to each well in duplicates. Untreated cells were included as vehicle control. Cells were then stained with 7‐aminoactinomycin D (7AAD) for viability assay. Cell viability results were normalized to vehicle controls and then inputted into the CalcuSyn program (http://www.biosoft.com/w/calcusyn.htm) that calculated each combination index (CI) value using the Chou and Talalay method, where CI < 1 indicates synergy, CI = 1 indicates additive effect and CI > 1 indicates antagonism.

### Animal studies

All experiments were performed under an Institutional Animal Care and Use Committee‐approved protocol, and institutional guidelines for the proper and human use of animals in research were followed.

#### AML cell lines and primary AML, syngeneic xenotransplantation assays

Six‐ to 8‐week‐old female nono‐obese diabetic/severe combined immunodeficiency (NOD/SCID) mice were purchased from Shanghai Ling Chang Biotechnology Co (Shanghai, P.R. China) under the approval of the institutional animal facility at Shanghai JiaoTong University Medical School. To investigate the effect of SIRT3 activity on tumourigenesis *in vivo*, xeno‐transplants were established by intravenous injection of 5 × 10^6^ of PCDH‐CMV‐MCS‐EF1‐coEGFP, wild type SIRT3 stably‐transfected AML cells per mouse into sub‐lethally irradiated (2·5 Gy) mice. Human AML engraftment was determined at 6–8 weeks post‐transplantation. To further explore the LSC activity after chemotherapy and synergize the effects of Ara‐C and the SIRT3 selective inhibitor 3‐TYP, xeno‐transplants were established as aforementioned. Following the development of AML (approximately 5 weeks post‐injection), the mice were treated with either PBS, Ara‐C (45–60 mg/kg), 3‐TYP (50 mg/kg) or combination of Ara‐C and 3‐TYP via intraperitoneal injection. Mice survival was monitored, and tumour burden was determined by flow cytometry.

#### Evaluation of AML engraftment

Animals were sacrificed and the bone marrow mononuclear cells (BMMCs) were collected, and the cells were stained with APC‐H7‐conjugated human CD45, APC‐conjugated human CD34 and PE‐Cy7‐conjugated human CD38 antibodies respectively, at room temperature for 15 min. Cells were then washed and re‐suspended in FACS buffer containing 7AAD (1:20 dilution), and analysed for the presence of viable human leukaemic cells (7AAD^−^/hCD34^+^/hCD38^−^).

### Statistical analyses

Unpaired student *T* test was utilized to compare the differences between two groups. Two‐way analysis of variance (ANOVA) was performed to compare the differences between three or more groups. Correlations between the viability of primary AML cells and acetylated SOD2 expression level or the production of total and mitochondria ROS were performed using linear regression analysis in GraphPad prism version 6. 01.

## Results

### SOD2 acetylation and mitochondrial ROS induction correlate with chemosensitivity in AML

**Figure 1:**
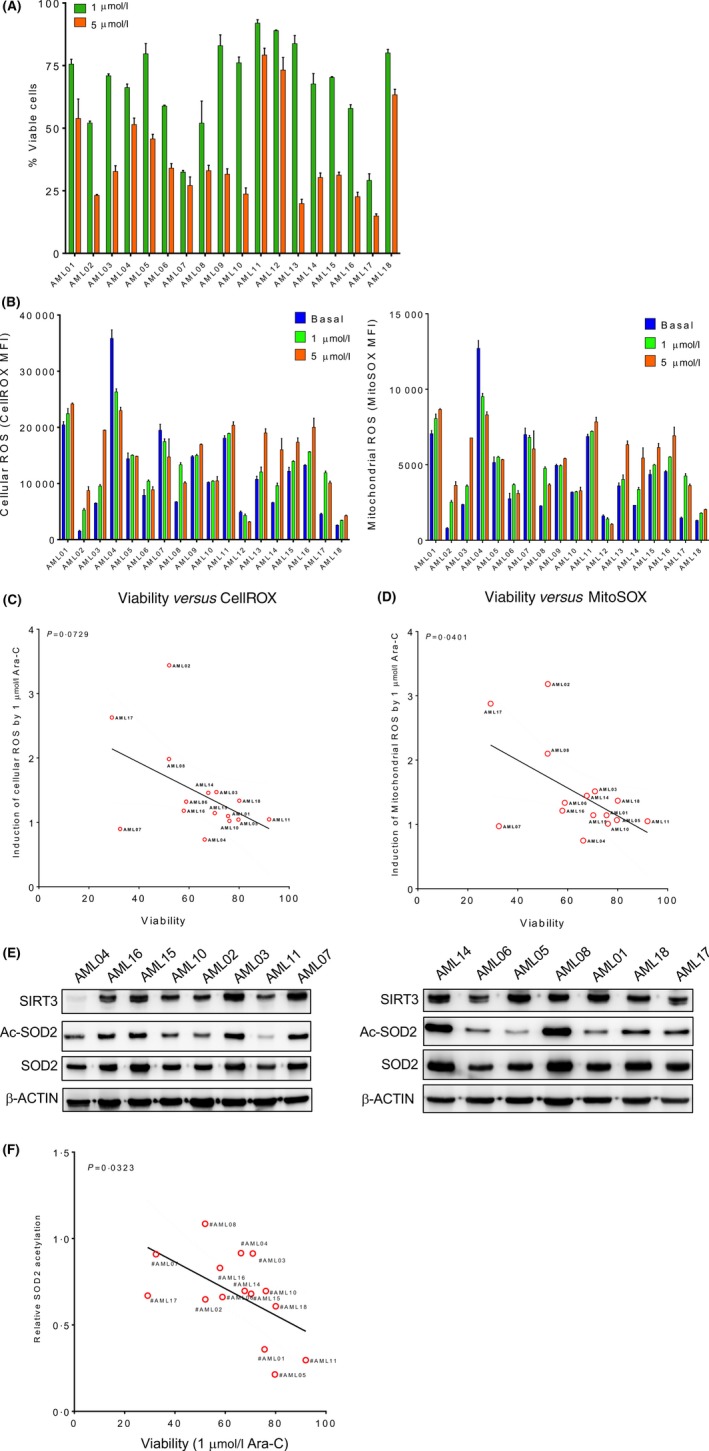
SOD2 acetylation associates with chemosensitivity. (A) Eighteen primary AML patient samples were treated with indicated doses of Ara‐C for 48 h. Cell viability was analysed by annexin V/7‐AAD staining and flow cytometry. (B) All primary AML patient samples were stained with CellROX and MitoSOX. The cellular and mitochondrial ROS levels were shown as MFI determined by flow cytometry. Data in (A, B) are representative of the mean ± standard deviation from technical triplicates (*n* = 18). The induction of cellular (C) and mitochondrial ROS (D) in primary AML samples treated with 1 μmol/l of Ara‐C compared to mock treated samples was evaluated for the correlation with response of these cells to 1 μmol/l of Ara‐C. (E) Cell lysates from 15 primary AML patient samples were analysed for SIRT3, total and acetylated SOD2 expression by Western blotting. β‐actin was probed as a loading control. (F) Relative expression of acetylated SOD2 in primary AML patient samples was determined based on total and acetylated SOD2 levels and was evaluated for the correlation with response of these primary cells to 1 μmol/l of Ara‐C. Data in (C), (D) and (F) are linear regression (*n* = 15). Ac‐SOD2, acetylated SOD2; AML, acute myeloid leukaemia; Ara‐C, cytarabine; MFI, mean fluorescence intensity; ROS, reactive oxygen species. [Colour figure can be viewed at http://wileyonlinelibrary.com]

To identify the key factors in mitochondria that are capable of regulating sensitivity of AML cells to chemotherapeutic agents, a total of 18 primary AML patient samples (Table 1) were subjected to Ara‐C treatment. All these samples showed variable sensitivity to Ara‐C (Fig 1A). The basal and Ara‐C induced total and mitochondrial ROS was stained by CellROX (Fig 1B, left panel) and MitoSOX (Fig 1B, right panel), respectively, and analysed via flow cytometry. Both basal and Ara‐C induced total and mitochondrial ROS showed similar staining profiles among these samples, suggesting that mitochondrial ROS is the major source of cellular ROS, and ROS induction is an important event for chemotherapies. The correlation between ROS and chemosensitivity in AML was then analysed. Notably, the sensitivity of these 18 primary patient AML cells to cytarabine was not correlated to the basal total and mitochondrial ROS level but showed a significant correlation to the induction of mitochondrial ROS (*P* = 0·0401, Fig 1C) with a lesser significance to the induction of total cellular ROS level (*P* = 0·0729, Fig 1D). We then explored the mechanism by which mitochondria regulate chemotherapeutic agent‐induced ROS production. Interestingly, among the key redox enzymes examined, our data showed that relative acetylation of SOD2, as determined by acetylated SOD2 normalized to total SOD2, correlated with the sensitivity of AML cells to cytarabine (Fig 1E, 1). These data suggest regulation of SOD2 acetylation in mitochondria may be the critical factor determining ROS induction by chemotherapeutic agents, and hence, contribute to chemoresistance in AML.

**Table 1:** Clinical characteristics of AML patients. The table provides details of the 18 primary AML cells analysed in our study.

Patient ID	Sex/age (years)	Disease status	WHO classification of AML^a^	Mutations
AML01	F/46	Relapse	AML‐MRC	None
AML02	M/31	*De novo*	AML, NOS	None
AML03	F/69	*De novo*	AML, NOS	None
AML04	F/30	*De novo*	AML‐MRC	None
AML05	F/65	*De novo*	AML, NOS	*KRAS*
AML06	M/58	*De novo*	AML, NOS	*DEK‐NUP214* (+151·47)
AML07	F/45	Relapse	AML, NOS	None
AML08	F/53	Relapse	AML with biallelic mutations of *CEBPA*	*KIT*, *CEBPA*, *TET2*
AML09	F/49	*De novo*	AML, NOS	None
AML10	M/54	*De novo*	AML, NOS	None
AML11	M/40	Relapse	AML, NOS	*ETV6‐ABL1* (+108·97%)
AML12	M/54	Relapse	AML‐MRC	None
AML13	F/53	Relapse	AML, NOS	None
AML14	M/72	*De novo*	AML, NOS	*CBFB‐MYH11*(+160·77%)
AML15	M/24	*De novo*	AML, NOS	*RUNX1*‐*RUNX1T1* (+287·98), *IDH*, *KRAS*, *TET2*
AML16	F/42	*De novo*	AML, NOS	*KIT*, *WTI1*
AML17	F/41	*De novo*	AML, NOS	*RUNX1*
AML18	M/54	Relapse	AML, NOS	None

AML, acute myeloid leukaemia; F, female; M, male; MRC, myelodysplasia‐related changes; NOS, not otherwise specified; WHO, World Health Organisation.

a According toArber *et al *(2016)

## SIRT3 alters the sensitivity to chemotherapeutic agents in AML cells

**Figure 2:**
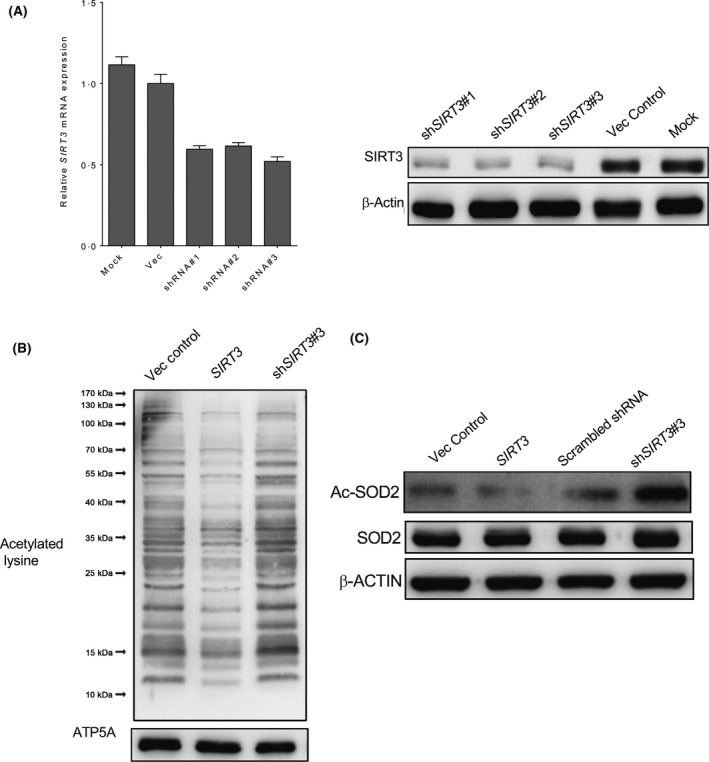
SOD2 acetylation is regulated by SIRT3 deacetylase. (A) MV4‐11 cells were transduced with three different shRNA lentiviral vectors targeting *SIRT3* alongside scrambled shRNA and mock control. Expression of *SIRT3* mRNA and SIRT3 protein was measured by quantitative polymerase chain reaction (left panel) and Western blotting (right panel). Data in (A) are representative of the mean ± standard deviation from technical triplicates. (B) Mitochondrial proteins from MV4‐11 cells transduced with empty vector, *SIRT3* and *SIRT3* shRNA were isolated, and analysed by sodium dodecyl sulphate‐polyacrylamide gel electrophoresis (SDS‐PAGE) and Western blotting using antibody against acetylated lysine. ATP5A was determined as the loading control. (C) Total cellular proteins obtained from MV4‐11 cells were separated on SDS‐PAGE and probed with antibodies against total and acetylated SOD2. β‐actin was included as a loading control. Ac‐SOD2, acetylated SOD2; Vec, vector.

It is well known that acetylation of SOD2, as well as a variety of other metabolic enzymes, is tightly regulated by the mitochondrial de‐acetylase, SIRT3. The role of SIRT3 in AML chemoresistance was investigated. However, similar to SOD2, there was no significant correlation between Ara‐C sensitivity and total SIRT3 protein levels in primary AML samples (Fig 1E), suggesting the certain active form of SIRT3 but not the total protein amount is the ultimate determinant for SOD2 post‐translational modification. Three *SIRT3* shRNA lentiviral vectors were used to knock down *SIRT3* in AML cells: all of them showed downregulation of *SIRT3* mRNA (Fig 2A, left panel) and SIRT3 protein levels (Fig 2A, right panel) as well as cell viability (Figure S1). To evaluate the impact of downregulation of SIRT3 deacetylase activity, MV4‐11 cells were transduced with lentivirus encoding vector control, *SIRT3* or *SIRT3*‐specific shRNA. The mitochondrial proteins were isolated and analysed for their pan‐acetylation using the antibody against acetylated lysine. Decreased mitochondrial pan‐acetylation was observed in *SIRT3* overexpressing AML cells. In contrast, the acetylation of mitochondrial proteins was upregulated by *SIRT3* knockdown (Fig 2B). As a result of *SIRT3* knockdown, SOD2 acetylation was increased (Fig 2C). As previously reported, these data indicated that SOD2 acetylation level is regulated by SIRT3.

**Figure 3:**
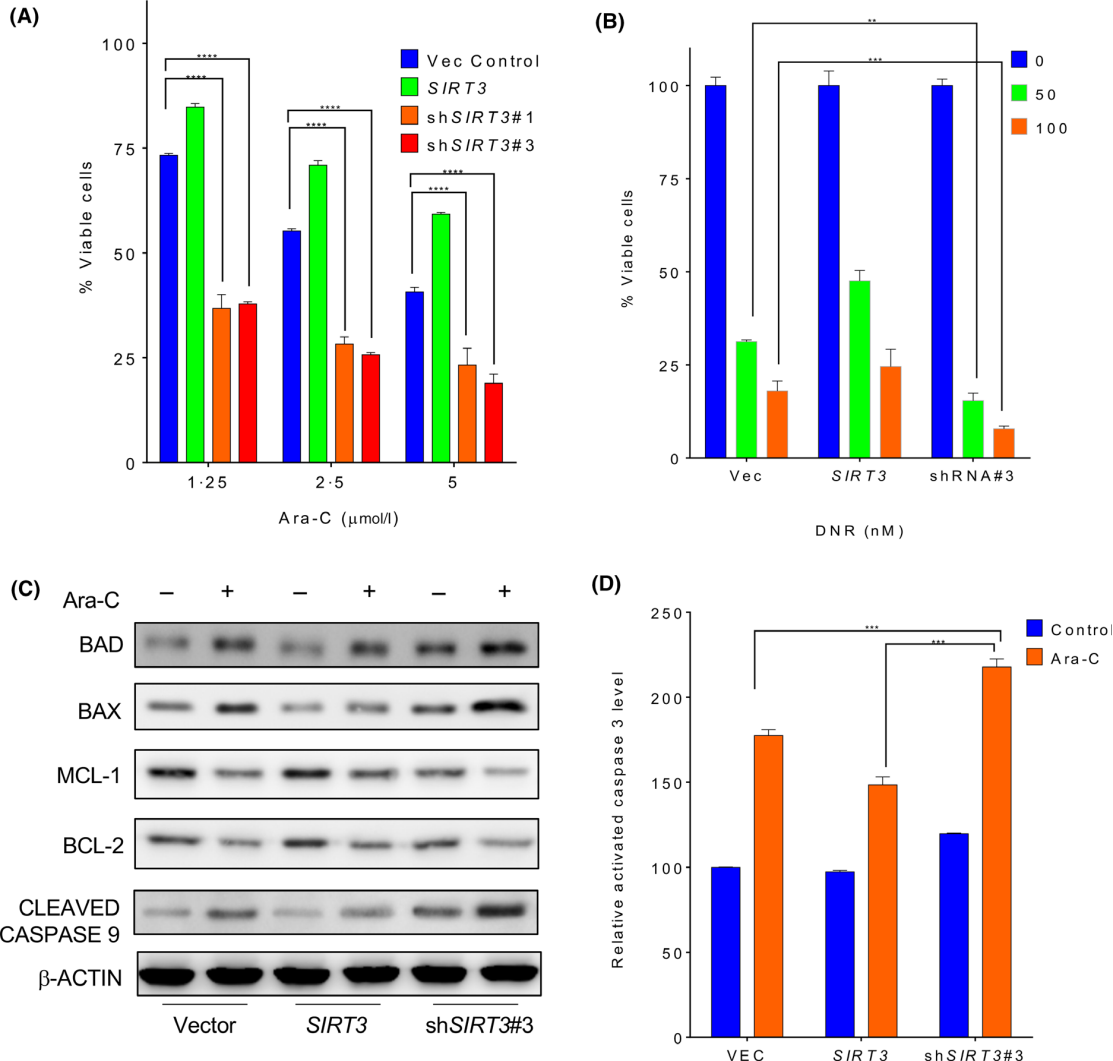
Increased SIRT3 deacetylase activity contributes to chemoresistance in AML cells. MV4‐11 cells transduced with empty vector, wild type *SIRT3*, *SIRT3* shRNA#1 (shSIRT3#1), and *SIRT3* shRNA#3 (shSIRT3#3) were treated with indicated doses of (A) cytarbine (Ara‐C) or (B) daunorubicin (DNR) for 48 h. Cell viability was analysed by annexin V/7‐AAD staining and flow cytometry. (C) MV4‐11 cells transduced with above indicated lentiviral plasmids were treated with 1 μmol/l Ara‐C for 24 h. Apoptosis related proteins including BAD, BAX, MCL1, BCL2 and cleaved caspase 9 were measured by Western blotting. β‐actin was used as a loading control. (D) Upon treatment with 1 μmol/l Ara‐C for 24 h, MV4‐11 cells were fixed, permeabilized and stained with Alexa Fluor 647‐conjugated antibody against active caspase 3 followed by flow cytometry analysis. Data in (A), (B) and (D) are representative of the mean ± standard deviation from technical triplicates. **P < 0·01; ***P < 0·005; ****P < 0·001, two‐way ANOVA. [Colour figure can be viewed at http://wileyonlinelibrary.com]

As mitochondria reprogramming is one of the critical events for chemoresistance, SIRT3 may be capable of regulating AML chemosensitivity via its deacetylase activity. The response of AML cells with *SIRT3* overexpression or knockdown to chemotherapeutic agents was determined. Compared to vector control, overexpression of *SIRT3* led to increased cell viability when treated with either Ara‐C (Fig 3A) or daunorubicin (Fig 3B) in all doses evaluated. In contrast, knockdown of *SIRT3* resulted in increased toxicity of these agents to AML cells, suggesting SIRT3 may contribute to AML chemoresistance. To confirm the apoptosis‐related subcellular changes affected by SIRT3, cellular proteins were extracted from Ara‐C treated MV4‐11 cells to analyse the alteration of pro‐ or anti‐apoptotic pathways. The induction of pro‐apoptotic BAD, BAX and cleaved caspase 9, as well as the reduction of anti‐apoptotic MCL1 and BCL1 by Ara‐C was neutralized in MV4‐11 cells that overexpressed *SIRT3* (Fig 3C), whereas *SIRT3* shRNA induced BAD, BAX and caspase 9, but downregulated MCL‐1 and BCL2 in Ara‐C treated cells (Figure S2). Furthermore, the activation of caspase 3 was evaluated using flow cytometry. Consistently, *SIRT3* knockdown significantly induced caspase 3 activation (Fig 3D). These data indicate that SIRT3 downregulation sensitizes AML cells to chemotherapeutic agents.

## SIRT3 regulates mitochondrial ROS in AML

**Figure 4:**
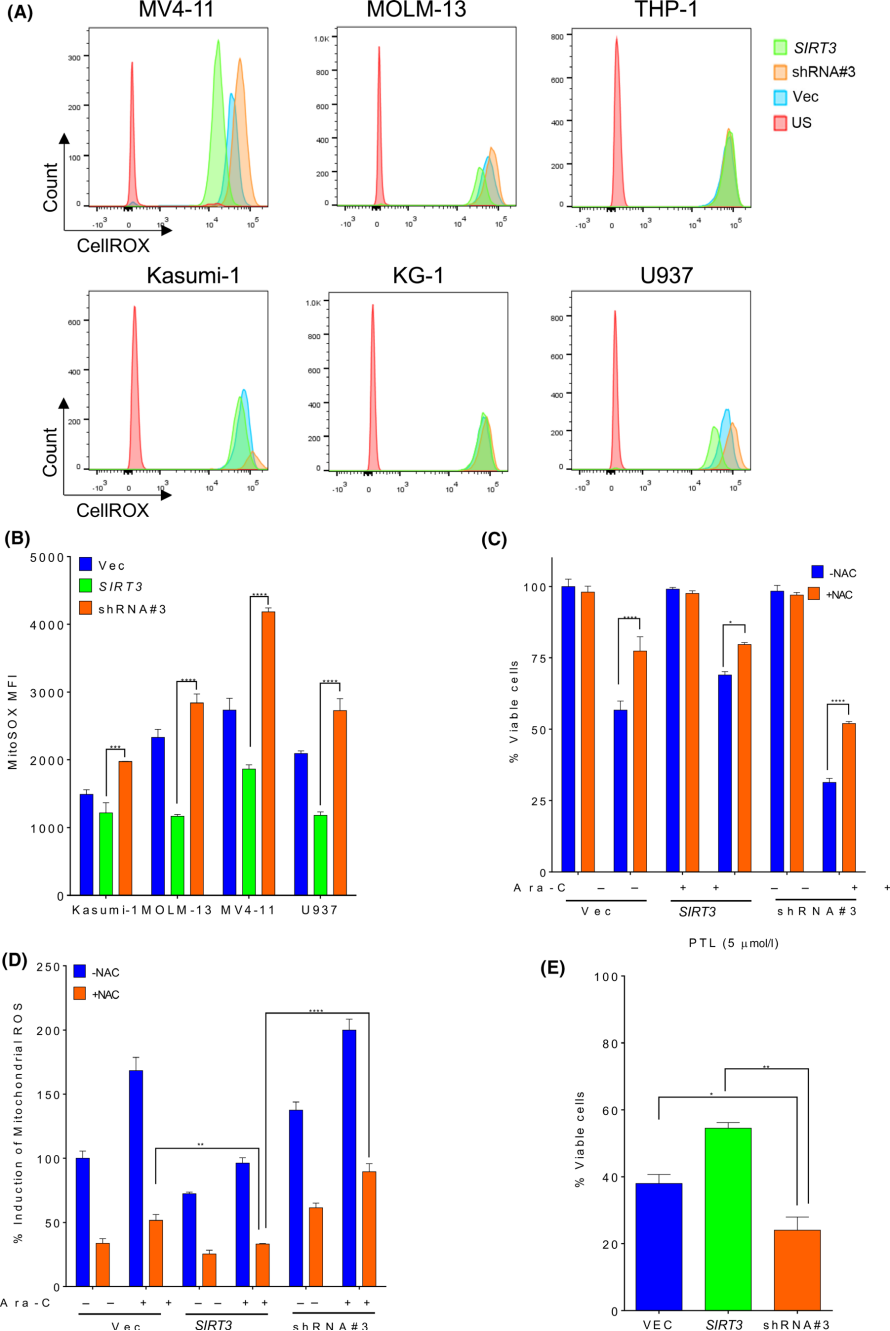
SIRT3 activity is associated with mitochondria ROS production in AML cells. (A) MV4‐11, MOLM‐13, THP‐1, Kasumi‐1, KG‐1, U937 and cells transduced with empty vector, wild type *SIRT3* or *SIRT3* shRNA#3 (shSIRT3#3) were stained with CellROX followed by flow cytometry analysis. Cellular ROS level was shown as histogram. (B) The level of mitochondrial ROS was determined in these cells by MitoSOX staining and flow cytometry analysis and shown as MFI. (C) MV4‐11 cells transduced with empty vector, *SIRT3* or shSIRT3#3 Cells were pre‐treated with 800 μmol/l of N‐acetyl cystine (NAC) for 1 h prior to exposure to 1 μmol/l Ara‐C. Cells were stained with annexin V/7‐AAD at 48 h post‐treatment and cell viability was analysed by flow cytometry. (D) MV4‐11 cells were pre‐treated with NAC prior to Ara‐C exposure at indicated dose. Mitochondrial ROS level in these cells was determined by MitoSOX staining and was expressed as percent induction normalized to mock treated vector control. (E) MV4‐11 cells transduced with empty vector, wild type *SIRT3* or shSIRT3#3 were treated with 10 μmol/l of parthenolide for 48 h. Cells were stained with annexin V/7‐AAD and analysed by flow cytometry. Data in (B–E) are representative of the mean ± standard deviation from technical triplicates. *P < 0·05, **P < 0·01, ***P < 0·005, ****P < 0·001, two‐way ANOVA. Ara‐C, cytarabine; MFI, mean fluorescence intensity; PTL, parthenolide; ROS, reactive oxygen species; Vec, vector. [Colour figure can be viewed at http://wileyonlinelibrary.com]

ROS is well known to regulate apoptosis in cancer cells and is critical for leukaemic stem and blast cells compared to their healthy counterparts. As an important mitochondrial protein modifier, SIRT3 is capable of deacetylating some key protein enzymes involved in the generation of mitochondrial ROS, such as SOD2. Thus, we examined the correlation between SIRT3 de‐SUMOylation induced chemoresistance with ROS in AML cells. Kasumi‐1 (*RUNX1‐RUNX1T1*), U‐937, KG‐1, THP‐1 (*KMT2A*‐MLLT3), MOLM‐13 [FLT3‐internal tandem duplication (ITD), *KMT2A*‐MLLT3] and MV4‐11 (FLT3‐ITD, *KMT2A*‐*AFF1*) cells were transduced with lentivirus encoding empty vector, wild type *SIRT3* or shRNA. The ROS level in these cells were measured by CellROX staining and flow cytometry. Among the six AML cell lines examined, four showed obvious decreased ROS levels when wild type *SIRT3* was overexpressed, including Kasumi‐1, U‐937, MOLM‐13 and MV4‐11 (Fig 4A). Furthermore, *SIRT3* shRNA transduced cells exhibited the highest ROS levels in the above‐mentioned AML cell lines. To verify whether change of ROS level in these cells was due to alteration of ROS production in mitochondria, where SIRT3 is predominantly located, cells were stained with MitoSOX and analysed by flow cytometry. Consistent with the total cellular ROS levels, *SIRT3* overexpression reduced mitochondrial ROS, which was significantly increased by its shRNA (Fig 4B). One of the key mechanisms that mediates chemotherapeutic agent‐induced AML cell apoptosis is their ability to upregulate cellular ROS. MV4‐11 cells were pre‐treated with the ROS scavenger, N‐acetyl cysteine (NAC), prior to exposure to Ara‐C. NAC prevented Ara‐C induced cell apoptosis in both control and *SIRT3* shRNA expressing AML cells, which was less obvious in SIRT3 overexpressing cells (Fig 4C). Accordingly, SIRT3 inhibited the ability of Ara‐C to induce mitochondria ROS, which was associated with acquired chemoresistance to the drug (Fig 4D), and was similar to the findings in the primary AML cells. To confirm that ROS is the key mechanism that SIRT3 utilises to regulate AML cell death upon drug treatment, MV4‐11 cells were exposed to PTL, a well‐known ROS inducer that displays selective toxicity to both leukaemic blasts and stem cells via ROS induction (Guzman *et al*, 2005). SIRT3 showed protection of AML cells against PTLbut, as expected, its knockdown sensitized AML cells to PTL (Fig 4E). These data suggest SIRT3 is capable of regulating AML sensitivity to chemotherapy via mitochondrial ROS production.

## SIRT3 regulates mitochondrial metabolism in AML

**Figure 5:**
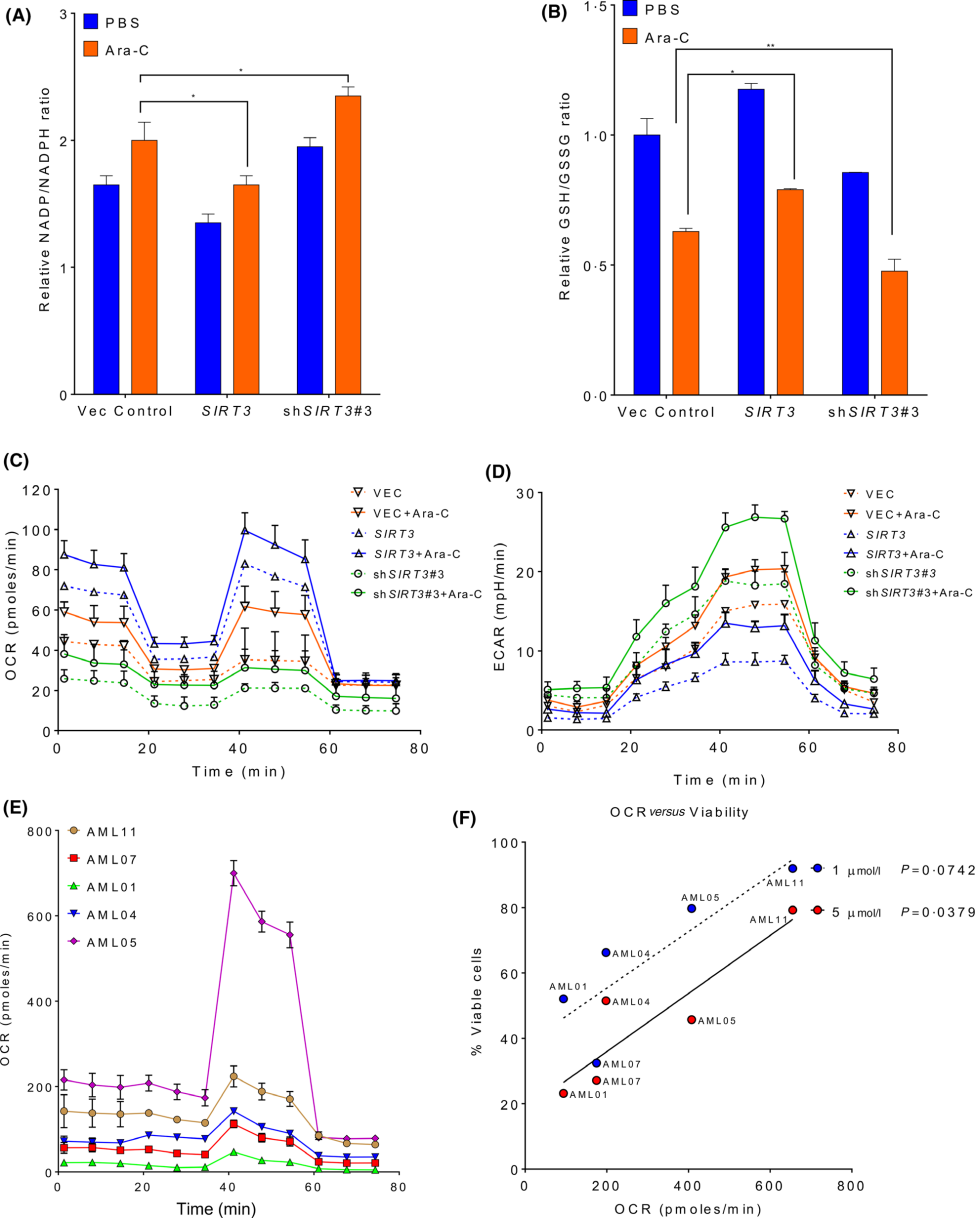
SIRT3 is capable of reprogramming mitochondrial metabolism. MV4‐11 cells transduced with vector control, *SIRT3* or wild type *SIRT3* shRNA#3 (shSIRT3#3) were treated either with PBS or 1 μmol/l of Ara‐C for 48 h. (A) Nicotinamide adenine dinucleotide phosphate (NADP)/reduced NADP (NADPH) and (B) reduced glutathione/oxidized glutathione (GSH/GSSG) ratios were analysed by the corresponding kits and absorbance. MV4‐11 cells transduced with above indicated lentiviral plasmids were treated either with (solid lines) or without (dash lines) 1 μmol/l of Ara‐C for 48 h. Cells were then seeded at the density of 1 × 10^6^/50 µl in a Cell Tak‐coated FX 96‐well plate and washed with base medium. OCR (C) and ECAR (D) were determined by Seahorse Agilent. (E) Primary AML cells were seeded at the density of 5 × 10^5^/50 µl in a pre‐coated 96 microplate. Cells were washed and resuspended in base medium, and OCR was determined by Seahorse Agilent. Data in (A–E) are representative of the mean ± standard deviation from technical triplicates. *P < 0·05, **P < 0·01, two‐way ANOVA. (F) Five primary AML cells were treated with 1 or 5 µmol/l Ara‐C for 48 h (*n* = 15). Cell viability was analysed by annexin V/7‐AAD staining and flow cytometry. The correlation between the percentage of primary cell viability and OCR were determined by linear regression analysis. OCR was significantly correlated with viability of 5 µmol/l Ara‐C (*P* = 0·0379) and was slightly less significantly correlated with cell viability of 1 µmol/l Ara‐C (*P* = 0·0742). AML, acute myeloid leukaemia; Ara‐C, cytarabine; ECAR, extracellular acidification rate; OCR, oxygen consumption rate; PBS, phosphate‐buffered saline; Vec, vector. [Colour figure can be viewed at http://wileyonlinelibrary.com]

Change in mitochondrial metabolism is a unique feature in bulk AML and LSCs. It is well known that SIRT3 is a crucial protein that is capable of regulating a variety of mitochondrial enzymes involved in metabolism via de‐acetylation. The impact of SIRT3 on AML cells was explored. As a major mitochondrial deacetylase, SIRT3 promotes the tricarboxylic acid (TCA) cycle and oxidative stress. We then determined if SIRT3 de‐SUMOylation conferred Ara‐C chemoresistance via the TCA cycle or oxidative stress. The NADP/NADPH ratio was lower in cells with *SIRT3* overexpression, even in the presence of Ara‐C treatment compared to cells transduced with vector control (Fig 5A). In contrast, *SIRT3* shRNA harbouring cells showed higher NADP/NADPH ratio, indicating reduced conversion of NADPH to NADP+ by oxidation. Similarly, *SIRT3‐*overexpressing AML cells contained a relatively high GSH/GSSG ratio with or without Ara‐C treatment, whereas *SIRT3* shRNA decreased GSH/GSSG ratio regardless of cytarabine treatment (Fig 5B). Given that SIRT3 impacts the biogenetic status of mitochondria, we further characterized if SIRT3 confers chemoresistance via regulation of mitochondrial activity. The basal oxygen consumption level was relatively high in *SIRT3*‐overexpressing AML cells, and was further increased upon Ara‐C treatment (Fig 5C). *SIRT3* knockdown showed the lowest oxygen consumption. Furthermore, glycolysis level was lowest in AML cells overexpressing SIRT3 and highest in *SIRT3* knockdown cells (Fig 5D), suggesting chemoresistance is closely related to high OxPhos status in AML cells. To confirm this, OxPhos status was determined in the available primary cells, as mentioned previously (Fig 5E). Indeed, among the 5 primary AML samples tested, those relatively resistant to Ara‐C generally displayed high OxPhos status, whereas the primary AML cells with the least active OxPhos were the most sensitive to chemotherapy (Fig 5F). Taken together, these data suggest SIRT3 confers Ara‐C chemoresistance via regulation of mitochondrial activity.

## SIRT3 inhibition synergizes with cytarabine in chemoresistant AML cells

**Figure 6:**
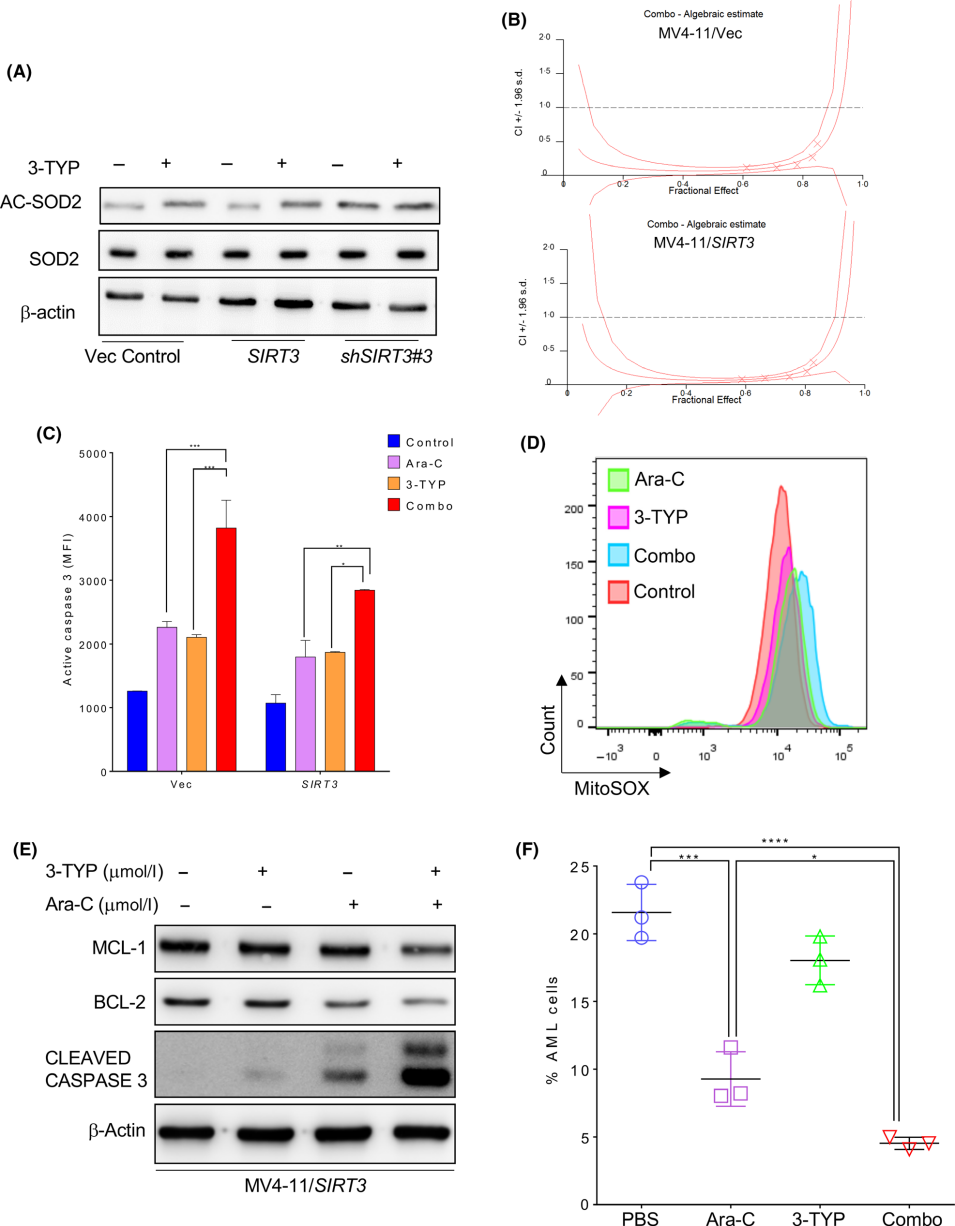
SIRT3 inhibitor synergizes with Ara‐C *in vitro* and in *vivo*. (A) MV4‐11 cells transduced with empty vector, wild type *SIRT3* or *SIRT3* shRNA#3 (shSIRT3#3) were treated with 50 μmol/l of 3‐TYP for 48 h. The whole cell lysates were probed for total and acetylated SOD2 as well as β‐actin. (B) MV4‐11/Vec or MV411/SIRT3 cells were treated with Ara‐C (0·5, 1, 2, 5, and 10 μmol/l) or 3‐TYP (5, 10, 20, 50 and 100 μmol/l) alone, or combined drugs with constant ratio (Ara‐C:3‐TYP = 1:10) for 48 h. Cell viability was determined by annexin V/7‐AAD staining and flow cytometry analysis. Synergistic effect between Ara‐C and 3‐TYP was shown as CI < 1. (C) MV4‐11/SIRT3 cells treated with Ara‐C or 3‐TYP alone or combination for 48 h. Cells were then permeabilized and stained with Alexa Fluor 647‐conjugated antibody against active caspase 3 followed by flow cytometry analysis. Data in (C) are representative of the mean ± standard deviation (SD) from technical triplicates *P < 0·05, **P < 0·01, ***P < 0·005, two way ANOVA. (D) MV4‐11/SIRT3 cells treated with Ara‐C or 3‐TYP alone or combination were stained with MitoSOX and shown as overlaid histogram. (E) Cell lysates from Ara‐C and/or 3‐TYP treated MV4‐11/SIRT3 cells were immunoblotted with antibodies against MCL‐1, BCL2 and cleaved caspase 3 as well as β‐actin. (F) MV4‐11/SIRT3 engrafted NOD/SCID mice were treated with Ara‐C or 3‐TYP alone or combination daily for 5 consecutive days. Seven days later, tumour burden was determined by percent GFP+ cells in the murine bone marrow. Data in (F) are representative of the mean ± SD from technical triplicates (*P < 0·05, ***P < 0·005, ****P < 0·001), one‐way ANOVA. 3‐TYP, 3‐(1H‐1,2,3‐triazol‐4‐yl) pyridine; Ara‐C, cytarabine; PBS, phosphate‐buffered saline. [Colour figure can be viewed at http://wileyonlinelibrary.com]

Induction of chemoresistance by SIRT3 provided the rationale of inhibiting SIRT3 to improve the anti‐leukaemic efficacy of chemotherapeutic agents. The SIRT3 selective inhibitor 3‐TYP [50% inhibitory concentrations (IC50s) of 16, 88 and 92 nmol/l for SIRT3, SIRT1 and SIRT3, respectively] was employed. Treatment with 3‐TYP indeed inhibited SIRT3 deacetylase activity as measured by SOD2 acetylation (Fig 6A). MV4‐11/Vec and MV4‐11/SIRT3 cells were treated with 0·5, 1, 2, 5 or 10 μmol/l of cytarabine alone, 5, 10, 20, 50 or 100 μmol/l of 3‐TYP alone, or in combination to evaluate the interaction between SIRT3 inhibition and chemotherapy. SIRT3 inhibition alone showed mild toxicity. However, synergism was observed in both MV4‐11/Vec and MV4‐11/SIRT3 cells when the treated with a constant ratio of Ara‐C:3‐TYP (1:10, Fig 6B). The synergism was further confirmed by flow cytometry analysis of activated caspase 3. Combination treatment with 3‐TYP and Ara‐C significantly induced caspase 3 activation (Fig 6C) and ROS level (Fig 6D) compared to Ara‐C or 3‐TYP alone. Similar changes to apoptosis‐related proteins, such as MCL1 and BCL2, were found (Fig 6E). To confirm these findings, NOD/SCID mice were engrafted with MV4‐11/*SIRT3*‐K288R. The mice were treated with Ara‐C or/and 3‐TYP. As a result, 3‐TYP alone slightly reduced AML in the mouse bone marrow. Combined treatment with 3‐TYP and cytarabine displayed the maximal anti‐leukaemic activity *in vivo* (Fig 6E). These data suggest SIRT3 may be a new target to enhance the potency of chemotherapeutic agents for a better clinical outcome.

## Discussion

Chemoresistance is the biggest challenge to therapeutics targeting AML. Increasing data indicate that mitochondria play a central role in the development of chemoresistance. Mitochondria are the major sites where the apoptotic signal cascades are initiated. In addition, mitochondrial pathways play other critical roles during tumourigenesis. For instance, mitochondrial ROS not only mediates the drug‐induced killing of AML blasts, but also regulates oncogene‐driving transformation, cancer stemness and apoptosis resistance (Bonnet *et al*, 2007; Weinberg *et al*, 2010; Janiszewska *et al*, 2012). OxPhos is involved in maintenance of cancer stemness, metastasis and drug resistance (Janiszewska *et al*, 2012; Roesch *et al*, 2013; LeBleu *et al*, 2014). Unlike the majority of solid tumours that are dependent on glycolysis to obtain ATP, known as the Warburg effect (Duvel *et al*, 2010), AML is primarily dependent on mitochondrial OxPhos for survival (Suganuma *et al*, 2010). AML cells exhibit a greater mitochondrial mass and higher rates of oxygen consumption compared to their normal counterparts (Boultwood *et al*, 1996), despite that LSCs have relatively less mitochondrial mass compared to AML blasts. Therefore, targeting mitochondria may be a promising strategy to ablate both leukaemic blasts and stem cells for a better therapeutic outcome. BCL2 inhibitor has shown significantly improved clinical outcome in elder AML patients ineligible for standard induction therapy (Pollyea *et al*, 2018; DiNardo *et al*, 2019), by altering the mitochondria activity in quiescent leukaemic stem cells, which are generally ROS‐low (Lagadinou *et al*, 2013).

Post‐translational modifications of mitochondrial proteins, including acetylation, succinylation, ADP‐ribosylation, phosphorylation and ubiquitinylation have been extensively studied in the last decade. It has been shown that such post‐translational modifications can alter the activity, stability and subcellular localization of mitochondrial proteins. SIRT3 is the most important deacetylase for mitochondrial proteins. Its substrates include the key proteins involved in OxPhos (Complexes I, II and V), fatty acid metabolism (ACSS2, LCAD and HMGCS2), carbohydrate metabolism (Cyclophilin D and pyruvate dehydrogenase), ROS detoxification (IDH2 and SOD2) and apoptosis (MPTP and OGG1) (Hofer & Wenz, 2014). Furthermore, post‐translational modification of SIRT3 has also been shown. SIRT3 enzymatic activity is enhanced upon phosphorylation of SIRT3 at Thr150/Ser159 by cyclin B1/CDK1 (Liu *et al*, 2015). Here, we demonstrated that SIRT3 deacetylase activity, as reflected by SOD2 acetylation but not its protein level, is associated with sensitivity of AML to chemotherapeutic agents including Ara‐C and daunorubicin, suggesting the existence of a subset of an active form of SIRT3, which may be due to its certain post‐translational modifications. SIRT3 deacetylase activity is indispensable for AML cells. Loss of *SIRT3* resulted in apoptosis of AML cells *in vitro*, and disability of the AML cells to engraft immune‐deficient mice *in vivo*. However, the activating post‐translational modifications and the corresponding modifiers of SIRT3 remain yet to be identified.

It has been shown that abnormalities in ROS regulation are important factors driving the pathogenesis of various diseases, including cancers. For example, an IDH2 mutant found in AML plays an essential role in the epigenetic changes via its oncogenic metabolite, 2‐hydroxyglutarate, that has critical impact on epigenetic changes in AML, as well as its capability to alter the mitochondrial ROS production via NADPH and GSH (Jo *et al*, 2001; Lee *et al*, 2003; Ward *et al*, 2010). Expression of haem oxygenase‐1 (HO‐1), a rate‐limiting enzyme of the haem catabolic process, can be induced by oncoproteins like FLT3 during leukemogenesis (Hornbaker *et al*, 2015). SIRT3‐mediated deacetylation often leads to the enhanced enzymatic activity of its targets, such as SOD2 and IDH2, and, as a consequence, the decreased mitochondrial ROS production. We showed that primary AML cells with higher resistance to the chemotherapeutic agents displayed lower induction ability of ROS regardless of basal ROS level, presumably because AML cells from different patients were accustomed to the basal ROS level due to individual variation, whereas the fold induction of ROS reflects the damage of these cells by the treatment, as demonstrated elsewhere (Halasi *et al*, 2013). Resistance of such primary AML cells reversely correlated with acetylation of SOD2, an event modulated by SIRT3. In addition, *SIRT3* overexpression in AML cells downregulated both basal and drug‐induced total and mitochondrial ROS levels, resulting in enhanced survival of leukaemic cells exposed to cytarabine *in vitro* and in xenograft mouse models. Consist with the previous report by Farge *et al *(2017), increased SIRT3 expression led to the transformation of metabolism in AML cells to enhanced OxPhos, a critical event in chemoresistance to Ara‐C. Similar results were obtained from the current study. On one hand, patient‐derived AML cells bearing higher OxPhos metabolism were more resistant to chemotherapy. On the other hand, SIRT3 mediated reprogramming of mitochondrial metabolism to OxPhos led to acquired resistance to chemotherapy. These data indicate SIRT3 is a critical mitochondrial factor regulating chemoresistance by reprogramming of mitochondrial OxPhos metabolism. De‐regulation of SIRT3 due to the abnormality of its post‐translational modification may have a significant impact on sensitivity of AML cells to chemotherapies through, at least in part, modulation of mitochondrial ROS production via metabolic enzymes.

The key regulators involved in the production of ROS can be utilized as new therapeutic targets for AML. For instance, NADPH oxidase‐2 (NOX2) is capable of regulating the mitochondrial transfer from bone marrow stromal cells (BMSC) to AML blasts, an event observed in a variety of cancers to potentiate survival of malignant cells. NOX2 inhibitor disabled mitochondrial transfer, increased AML cell apoptosis and improved survival of xenograft mice (Marlein *et al*, 2017). A synergy was observed between metformin, which inhibits Complex I in mitochondria, and the broad‐spectrum metabolic inhibitor 6‐benzylthioinosine (6‐BT), with reduced glycolysis, ROS suppression and increased apoptosis (Sabnis *et al*, 2016). Inhibition of HIF1α by 2‐methoxyestradiol was found to activate the mitochondrial apoptotic pathway in AML (Zhe *et al*, 2016). Inhibitors for histone deacetylases as well as protein deacetylase SIRT1 have been evaluated in clinical trials in the US and European Union. In comparison, targeting mitochondria by inhibition of SIRT3 has been poorly studied in clinical applications. In the current study, we demonstrated that 3‐TYP, a selective inhibitor for SIRT3, has minor impact on cell apoptosis alone. However, combination of 3‐TYP and Ara‐C eliminated the chemoresistance in AML cells by inhibiting SIRT3 deacetylase activity, inducing mitochondrial ROS generation, altering mitochondrial activity, and activating apoptotic signal cascades. A significantly improvement of the *in vivo* anti‐leukaemic efficacy was observed in mice treated with the combination of 3‐TYP and Ara‐C. In addition, sensitizing AML cells to chemotherapy by SIRT3 inhibitor may also be due to the altered unique biological function of its targets, such as IDH2.These data suggest SIRT3 and its modifiers can be exploited as therapeutic targets to ameliorate the anti‐tumour efficacy of currently used chemotherapeutic agents.

Taken together, our study demonstrated that SIRT3 deacetylase activity is associated with higher reducing and OxPhos status of the mitochondria, and dampened Ara‐C‐induced apoptosis as well as attenuated sensitivity of cell line‐derived xenograft mice to chemotherapy. Targeting mitochondria by inhibiting SIRT3 deacetylase activity may be a promising strategy to overcome chemoresistance of AML to standard induction therapies.

## Disclosure of Conflicts of Interest

There are no competing financial interests to declare.

## Authorship Contributions

JM conceptualized and directed the project, designed and performed the experiments, analysed the data and wrote the manuscript; BL collected clinical samples and analysed some of the data; DY performed some of the experiments, YZ shared valuable opinions on data presentation; YC contributed essential research discussion; YJM coordinated clinical resources and designed the project; JKC conceptualized the project. All authors have read and approved the manuscript.

## Supporting information


**Fig S1.** SIRT3 is essential for AML cells survival.
**Fig S2.** Increased SIRT3 deacetylase activity contributes to chemoresistance in AML cells.

**Data S1.** Increased SIRT3 deacetylase activity contributes to chemoresistance in AML cells.
