# Supplementary figures and images for: SIRT3 deacetylase activity confers chemoresistance in AML via regulation of mitochondrial oxidative phosphorylation
Source: Br J Haematol. 2019 Jun 24;187(1):49–64. doi: 10.1111/bjh.16044 (PMC6790595; doi:10.1111/bjh.16044)

### Supplementary Figure 1.

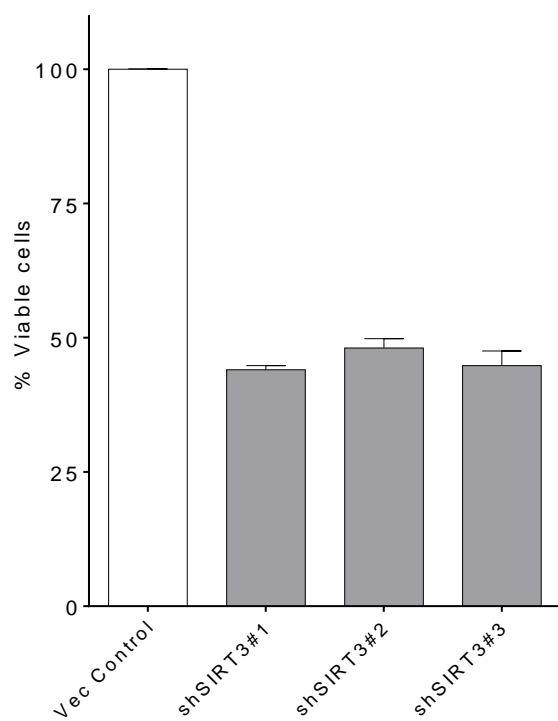

**Supplementary Figure 2.**

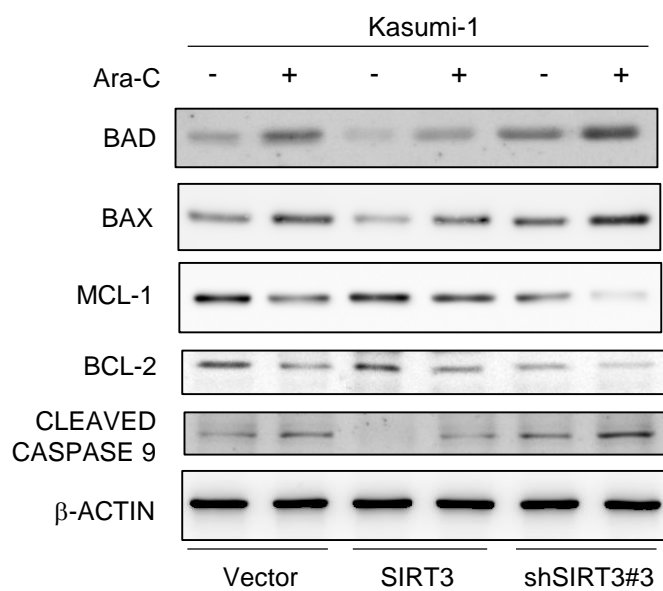

Supplement: Supplementary file 1 — Fig S1. SIRT3 is essential for AML cells survival. Fig S2. Increased SIRT3 deacetylase activity contributes to chemoresistance in AML cells. [file BJH-187-49-s001.pdf]
