# Supplementary material for: SIRT3 deacetylase activity confers chemoresistance in AML via regulation of mitochondrial oxidative phosphorylation
Source: Br J Haematol. 2019 Jun 24;187(1):49–64. doi: 10.1111/bjh.16044 (PMC6790595; doi:10.1111/bjh.16044)
Supplement: Supplementary file 2 — Data S1. Increased SIRT3 deacetylase activity contributes to chemoresistance in AML cells. [file BJH-187-49-s002.pdf]

Supplementary materials and methods

Human SIRT3 and shSIRT3 plasmids construction

Construction of human SIRT3 plasmid was performed by using hsSIRT3-F: AATTCTAGAATGGCGTTCTGGGGTTGGCGCGC, and hsSIRT3-R: CGCGAATTCCTATTTGTCTGGTCCATCAAGCTTCC primer set. The DNA fragment was then gel purified and restriction digested with XbaI and EcoRI prior to cloned into PCDH-CMV-MCS-EF1-coEGFP. Human shSIRT3#1, 2 and 3 were PCR amplified using primer sets: shSIRT3#1-F: GATCCCCAACGTCACTCACTACTTTCCTTCCTGTCAGAGAAAGTAGTGAGTGAC GTTGGTTTTTG, shSIRT3#1-R: AATTCAAAAACCAACGTCACTCACTACTTTCTCTGACAGGAAGGAAAGTAGTGAG TGACGTTGGG; shSIRT3#2-F: GATCCTGGGCAGGTGAAACCAGAATACTTCCTGTCAGATATTCTGGTTTCACCTG CCCATTTTTG, shSIRT3#2-R: AATTCAAAAATGGGCAGGTGAAACCAGAATATCTGACAGGAAGTATTCTGGTTTC ACCTGCCCAG; shSIRT3#3-F: GATCCGGCATTTGTCCCTGGATTTATCTTCCTGTCAGAATAAATCCAGGGACAAAT GCCTTTTTG, shSIRT3#3-R: AATTCAAAAAGGCATTTGTCCCTGGATTTATTCTGACAGGAAGATAAATCCAGGGA CAAATGCCG, respectively. The DNA fragment was then gel purified and double digested with Encore and BamHIII prior to sub-cloning into the pGreenPuro shRNA

Cloning and Expression (System Biosciences Inc. Palo Alto, CA).

Lentiviral packaging and transduction

Lentivirus were produced after transient transfection of HEK 293T cells with the individual lentiviral plasmid along with the packaging plasmids (VSV.G, CMV R8.74). Empty lentiviral expression vector was also included as a vehicle control. The supernatant containing viral particles were collected 24h and 48h post transfections and enriched with lenti-X concentrator (Clontech) according to manufacturer's instruction. In brief, 1 volume of Lenti-X concentrator was combined with 3 volumes of viral supernatant and mixed by gentle inverting 10 times. Mixture was then centrifuged at 1500g for 45 min at 4 ^{o}C. Pellets were resuspended in 1/100 of the original volume using serum and antibiotic free medium. Lentivirus were then allocated and frozen at -80 ^{o}C until further usage. AML cells were then transduced with lentiviruses in a 6 well pre-coated with 20ug/ml Novonectin, viral particles were transduced by co-centrifugation at 2400 rpm for 90 min at room temperature in the presence of 8μg/ml polybrene.

Immunoblotting assay

Immunoblotting was performed according to a previously described method (Ma*, et al* 2014) Intensity of acetylated SOD protein was measured by grayscale using Image J software and normalized to total SOD2 expression.

Immunoprecipitation

Immunoprecipitation was performed according to a previously described method (Zong, *et al* 2005).

Cell viability determination

Primary AML samples or AML cells were treated with Ara-C, Danorubicin, Parthenolide (PTL) at the indicated doses respectively, for up to 72 h. For primary AML samples, cells were stained with CD34, CD38 and CD45 (BD pharmingen) prior to Annexin V/7-aminoactinomycin (7AAD, Thermofisher scientific) double staining. At least $1x10^5$ events for primary AML cells or $2x10^4$ events for AML cells lines were collected under each condition.

References

Ma, J., Lu, P., Guo, A., Cheng, S., Zong, H., Martin, P., Coleman, M. & Wang, Y.L. (2014) Characterization of ibrutinib-sensitive and -resistant mantle lymphoma cells. *Br J Haematol,* **166,** 849-861.

Zong, H., Li, Z., Liu, L., Hong, Y., Yun, X., Jiang, J., Chi, Y., Wang, H., Shen, X., Hu, Y., Niu, Z. & Gu, J. (2005) Cyclin-dependent kinase 11(p58) interacts with HBO1 and enhances its histone acetyltransferase activity. *FEBS Lett,* **579,** 3579-3588.
