# Supplementary material for: SIRT3 deacetylase activity confers chemoresistance in AML via regulation of mitochondrial oxidative phosphorylation
Source: Br J Haematol. 2019 Jun 24;187(1):49–64. doi: 10.1111/bjh.16044 (PMC6790595; doi:10.1111/bjh.16044)
Supplement: Supplementary file 3 [file BJH-187-49-s003.pdf]

Supplemental Figure 1. **SIRT3 is essential for AML cells survival.** MV4-11 cells transduced with empty vector, SIRT3 shRNA#1. shRNA#2 and shRNA#3 were treated with 1 μM Ara-C for 24h. Cell viability was analysed by annexin V/7-AAD.

Supplemental Figure 2. **Increased SIRT3 deacetylase activity contributes to chemoresistance in AML cells**. Kasumi-1 cells transduced with empty vector, wild type SIRT3, and shSIRT3#3 were treated with 1 μM of Ara-C for 24 h. Apoptosis related proteins including BAD, BAX, MCL-1, BCL-2 and cleaved caspase 9 were measured by Western blotting. β-actin was used as a loading control.
